# 1,2-Bis(4-methyl­benz­yl)diselane

**DOI:** 10.1107/S1600536812007726

**Published:** 2012-03-07

**Authors:** Mei-Yun Zhou, Yi-Qun Li, Wen-Jie Zheng

**Affiliations:** a Department of Chemistry, Jinan University, Guangzhou 510632, People’s Republic of China

## Abstract

The title mol­ecule, C_16_H_18_Se_2_, features a diselenide bridge between two 4-methyl­benzyl units, in which the central C—Se—Se—C torsion angle is 88.1 (3)°, while the two Se—Se—C—C fragments assume *gauche* conformations, with torsion angles of −51.8 (5) and 59.1 (4)°. The dihedral angle between the benzene rings is 78.9 (2)°.

## Related literature

For applications of organoselenium compounds, see: Garud *et al.* (2007 ▶). For the synthesis of the title compound, see: Saravanan *et al.* (2003 ▶); Zhou *et al.* (2011 ▶). For related structures, see: Hua *et al.* (2010 ▶); Liu *et al.* (2006 ▶); Zhou *et al.* (2011 ▶).
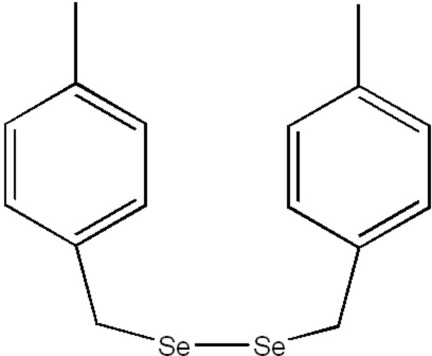


## Experimental

### 

#### Crystal data

C_16_H_18_Se_2_
*M* *_r_* = 368.22
Monoclinic, 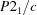

*a* = 5.8748 (7) Å
*b* = 11.5315 (11) Å
*c* = 22.794 (3) Å
β = 91.701 (9)°
*V* = 1543.5 (3) Å^3^
*Z* = 4
Mo *K*α radiation
μ = 4.77 mm^−1^
*T* = 293 K
0.40 × 0.09 × 0.09 mm

#### Data collection

Agilent Xcalibur Sapphire3 Gemini Ultra diffractometer
Absorption correction: multi-scan (*CrysAlis PRO*; Agilent, 2010 ▶) *T*_min_ = 0.469, *T*_max_ = 1.000
4989 measured reflections
2708 independent reflections
1806 reflections with *I* > 2σ(*I*)
*R*_int_ = 0.041

#### Refinement

*R*[*F* ^2^ > 2σ(*F* ^2^)] = 0.051
*wR*(*F* ^2^) = 0.111
*S* = 1.02
2708 reflections
165 parameters
H-atom parameters constrained
Δρ_max_ = 0.39 e Å^−3^
Δρ_min_ = −0.91 e Å^−3^

### 

Data collection: *CrysAlis PRO* (Agilent, 2010 ▶); cell refinement: *CrysAlis PRO*; data reduction: *CrysAlis PRO*; program(s) used to solve structure: *SHELXS97* (Sheldrick, 2008 ▶); program(s) used to refine structure: *SHELXL97* (Sheldrick, 2008 ▶); molecular graphics: *SHELXTL* (Sheldrick, 2008 ▶); software used to prepare material for publication: *publCIF* (Westrip, 2010 ▶).

## Supplementary Material

Crystal structure: contains datablock(s) I, global. DOI: 10.1107/S1600536812007726/bh2412sup1.cif

Structure factors: contains datablock(s) I. DOI: 10.1107/S1600536812007726/bh2412Isup2.hkl

Supplementary material file. DOI: 10.1107/S1600536812007726/bh2412Isup3.cml

Additional supplementary materials:  crystallographic information; 3D view; checkCIF report

## Figures and Tables

**Fig. 1.**
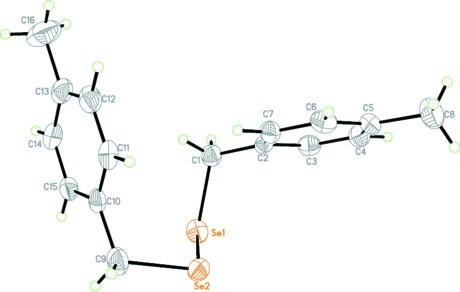
The molecular structure of the title compound showing displacement ellipsoids at the 30% probability level.

## supplementary crystallographic information

### Comment

Organoseleniums have been synthesized as anticancer agent, and for other medicinal applications, as well as biologically active substances exhibiting antiviral, antibacterial, antihypertensive, and fungicidal properties (Garud *et al.*, 2007). In continuation of our work on the synthesis and structures of derivatives of selenium (Zhou *et al.*, 2011), the title compound was prepared and its crystal structure is reported.

The title molecule (Fig. 1) features a diselenide bridge between two 4-methylbenzyl units. The central C—Se—Se—C torsion angle is 88.1 (3)°, while the two Se—Se—C—C fragments assume *gauche* conformations with values of -51.8 (5) and 59.1 (4)°. The dihedral angle between the two benzene rings is 78.9 (2)°. All bond lengths and angles are similar to those found in related structures (Hua *et al.*, 2010; Liu *et al.*, 2006; Zhou *et al.*, 2011).

### Experimental

Sodium borohydride (0.95 g, 25 mmol) was added to a vigorously stirred mixture of selenium powder (2.00 g, 25 mmol) and water (50 ml) at 0°C, then warmed to room temperature and stirred for 2 h. 1-(Bromomethyl)-4-methylbenzene (4.62 g, 25 mmol) was added to the mixture and stirred for 2 h. O_2_ was passed through the solution slowly, for 2 h (Saravanan *et al.*, 2003; Zhou *et al.*, 2011). The mixture was extracted with ethyl acetate (200 ml) and washed three times with water (50 ml × 3) and dried over anhydrous sodium sulfate. The organic residue was further purified by silica gel column using dichloromethane as eluent. The solvent was then evaporated under vacuum and the solid residue was recrystallized from CH_3_OH to afford yellow crystals of the title compound (yield: 3.73 g, 80.2%).

### Refinement

Carbon-bound H atoms were positioned geometrically and treated as riding on their C atoms, with C—H distances of 0.93Å (aromatic), 0.97 (CH_2_) and 0.96 Å (CH_3_), and were refined with *U*_iso_(H)=1.2*U*_eq_(C) for CH and CH_2_ groups, and *U*_iso_(H)=1.5*U*_eq_(C) for the methyl groups.

### Crystal data

**Table:**

C_16_H_18_Se_2_	*F*(000) = 728
*M**_r_* = 368.22	*D*_x_ = 1.585 Mg m^−^^3^
Monoclinic, *P*2_1_/*c*	Mo *K*α radiation, λ = 0.7107 Å
Hall symbol: -P 2ybc	Cell parameters from 933 reflections
*a* = 5.8748 (7) Å	θ = 3.2–29.4°
*b* = 11.5315 (11) Å	µ = 4.77 mm^−^^1^
*c* = 22.794 (3) Å	*T* = 293 K
β = 91.701 (9)°	Prism, yellow
*V* = 1543.5 (3) Å^3^	0.40 × 0.09 × 0.09 mm
*Z* = 4	

### Data collection

**Table:**

Agilent Xcalibur Sapphire3 Gemini Ultra diffractometer	2708 independent reflections
Radiation source: Enhance (Mo) X-ray Source	1806 reflections with *I* > 2σ(*I*)
Graphite monochromator	*R*_int_ = 0.041
Detector resolution: 16.0288 pixels mm^-1^	θ_max_ = 25.0°, θ_min_ = 3.2°
ω scans	*h* = −4→6
Absorption correction: multi-scan (*CrysAlis PRO*; Agilent, 2010)	*k* = −7→13
*T*_min_ = 0.469, *T*_max_ = 1.000	*l* = −27→26
4989 measured reflections	

### Refinement

**Table:**

Refinement on *F*^2^	Primary atom site location: structure-invariant direct methods
Least-squares matrix: full	Secondary atom site location: difference Fourier map
*R*[*F*^2^ > 2σ(*F*^2^)] = 0.051	Hydrogen site location: inferred from neighbouring sites
*wR*(*F*^2^) = 0.111	H-atom parameters constrained
*S* = 1.02	*w* = 1/[σ^2^(*F*_o_^2^) + (0.04*P*)^2^] where *P* = (*F*_o_^2^ + 2*F*_c_^2^)/3
2708 reflections	(Δ/σ)_max_ = 0.001
165 parameters	Δρ_max_ = 0.39 e Å^−^^3^
0 restraints	Δρ_min_ = −0.91 e Å^−^^3^
0 constraints	

### Fractional atomic coordinates and isotropic or equivalent isotropic displacement parameters (Å^2^)

**Table:**

	*x*	*y*	*z*	*U*_iso_*/*U*_eq_	
Se1	0.12002 (10)	0.36751 (6)	0.23758 (3)	0.0552 (2)	
Se2	0.50760 (10)	0.39377 (5)	0.24250 (3)	0.0556 (2)	
C10	0.4109 (9)	0.5942 (5)	0.1691 (3)	0.0429 (14)	
C6	0.5077 (10)	0.1399 (6)	0.0874 (3)	0.0559 (17)	
H6	0.6441	0.1495	0.0683	0.067*	
C2	0.1859 (9)	0.2269 (5)	0.1334 (2)	0.0403 (14)	
C7	0.3887 (9)	0.2363 (5)	0.1049 (2)	0.0476 (15)	
H7	0.4468	0.3096	0.0973	0.057*	
C3	0.1048 (9)	0.1154 (5)	0.1433 (3)	0.0500 (16)	
H3	−0.0323	0.1057	0.1620	0.060*	
C15	0.2026 (9)	0.6475 (5)	0.1722 (3)	0.0479 (15)	
H15	0.1437	0.6645	0.2087	0.057*	
C1	0.0609 (9)	0.3312 (5)	0.1532 (3)	0.0514 (16)	
H1A	−0.1012	0.3190	0.1465	0.062*	
H1B	0.1051	0.3973	0.1298	0.062*	
C9	0.5378 (10)	0.5602 (5)	0.2243 (3)	0.0638 (19)	
H9A	0.4812	0.6053	0.2567	0.077*	
H9B	0.6977	0.5787	0.2204	0.077*	
C13	0.1619 (11)	0.6539 (5)	0.0667 (3)	0.0590 (18)	
C5	0.4281 (10)	0.0295 (5)	0.0978 (3)	0.0505 (16)	
C14	0.0798 (9)	0.6759 (5)	0.1216 (3)	0.0528 (17)	
H14	−0.0619	0.7109	0.1246	0.063*	
C11	0.4962 (10)	0.5722 (5)	0.1137 (3)	0.0530 (17)	
H11	0.6380	0.5373	0.1106	0.064*	
C4	0.2230 (10)	0.0197 (5)	0.1258 (3)	0.0552 (17)	
H4	0.1642	−0.0536	0.1330	0.066*	
C12	0.3736 (12)	0.6015 (5)	0.0636 (3)	0.0617 (18)	
H12	0.4334	0.5860	0.0271	0.074*	
C8	0.5624 (11)	−0.0765 (6)	0.0797 (3)	0.084 (2)	
H8A	0.4714	−0.1225	0.0529	0.126*	
H8B	0.6022	−0.1217	0.1139	0.126*	
H8C	0.6986	−0.0523	0.0610	0.126*	
C16	0.0258 (13)	0.6855 (7)	0.0112 (3)	0.106 (3)	
H16A	−0.1074	0.6374	0.0079	0.159*	
H16B	0.1179	0.6735	−0.0223	0.159*	
H16C	−0.0191	0.7654	0.0130	0.159*	

### Atomic displacement parameters (Å^2^)

**Table:**

	*U*^11^	*U*^22^	*U*^33^	*U*^12^	*U*^13^	*U*^23^
Se1	0.0591 (4)	0.0521 (4)	0.0554 (4)	−0.0040 (3)	0.0163 (3)	−0.0017 (3)
Se2	0.0581 (4)	0.0471 (4)	0.0609 (5)	0.0025 (3)	−0.0119 (3)	0.0037 (4)
C10	0.038 (3)	0.028 (3)	0.063 (4)	−0.004 (3)	−0.003 (3)	−0.001 (3)
C6	0.051 (4)	0.067 (4)	0.049 (4)	−0.005 (4)	0.004 (3)	−0.005 (4)
C2	0.041 (3)	0.037 (3)	0.042 (4)	−0.005 (3)	−0.012 (3)	0.005 (3)
C7	0.054 (4)	0.039 (3)	0.049 (4)	−0.005 (3)	0.002 (3)	0.000 (3)
C3	0.041 (3)	0.058 (4)	0.051 (4)	−0.006 (3)	−0.003 (3)	0.000 (3)
C15	0.057 (4)	0.036 (3)	0.052 (4)	0.001 (3)	0.010 (3)	−0.004 (3)
C1	0.047 (3)	0.047 (4)	0.060 (4)	0.003 (3)	−0.006 (3)	−0.002 (3)
C9	0.063 (4)	0.042 (4)	0.086 (5)	−0.010 (3)	−0.009 (4)	−0.004 (4)
C13	0.063 (4)	0.045 (4)	0.069 (5)	−0.016 (4)	−0.008 (4)	0.013 (4)
C5	0.060 (4)	0.046 (4)	0.044 (4)	0.003 (3)	−0.004 (3)	−0.009 (3)
C14	0.044 (3)	0.040 (4)	0.075 (5)	0.003 (3)	0.007 (3)	0.011 (4)
C11	0.051 (4)	0.035 (4)	0.074 (5)	0.001 (3)	0.015 (3)	0.002 (4)
C4	0.063 (4)	0.044 (4)	0.058 (5)	−0.010 (3)	−0.007 (3)	0.002 (4)
C12	0.082 (5)	0.046 (4)	0.058 (5)	−0.009 (4)	0.023 (4)	0.000 (4)
C8	0.098 (6)	0.061 (5)	0.092 (6)	0.010 (4)	0.001 (4)	−0.021 (5)
C16	0.113 (6)	0.127 (7)	0.077 (6)	−0.014 (6)	−0.031 (5)	0.034 (6)

### Geometric parameters (Å, º)

**Table:**

Se1—Se2	2.2964 (9)	C9—H9A	0.9700
Se1—C1	1.989 (6)	C9—H9B	0.9700
Se2—C9	1.973 (6)	C13—C14	1.377 (9)
C10—C15	1.373 (7)	C13—C12	1.386 (9)
C10—C9	1.496 (8)	C13—C16	1.521 (8)
C10—C11	1.396 (8)	C5—C4	1.385 (8)
C6—H6	0.9300	C5—C8	1.519 (8)
C6—C7	1.379 (7)	C14—H14	0.9300
C6—C5	1.379 (8)	C11—H11	0.9300
C2—C7	1.378 (7)	C11—C12	1.373 (8)
C2—C3	1.392 (7)	C4—H4	0.9300
C2—C1	1.487 (7)	C12—H12	0.9300
C7—H7	0.9300	C8—H8A	0.9600
C3—H3	0.9300	C8—H8B	0.9600
C3—C4	1.369 (8)	C8—H8C	0.9600
C15—H15	0.9300	C16—H16A	0.9600
C15—C14	1.383 (8)	C16—H16B	0.9600
C1—H1A	0.9700	C16—H16C	0.9600
C1—H1B	0.9700		
			
C1—Se1—Se2	102.67 (16)	H9A—C9—H9B	107.8
C9—Se2—Se1	102.31 (17)	C14—C13—C12	117.7 (6)
C15—C10—C9	119.7 (6)	C14—C13—C16	121.4 (6)
C15—C10—C11	118.2 (5)	C12—C13—C16	120.9 (7)
C11—C10—C9	122.0 (5)	C6—C5—C4	117.3 (6)
C7—C6—H6	119.4	C6—C5—C8	121.0 (6)
C7—C6—C5	121.1 (6)	C4—C5—C8	121.7 (6)
C5—C6—H6	119.4	C15—C14—H14	119.1
C7—C2—C3	117.0 (5)	C13—C14—C15	121.8 (6)
C7—C2—C1	121.4 (5)	C13—C14—H14	119.1
C3—C2—C1	121.6 (5)	C10—C11—H11	119.6
C6—C7—H7	119.1	C12—C11—C10	120.9 (6)
C2—C7—C6	121.7 (5)	C12—C11—H11	119.6
C2—C7—H7	119.1	C3—C4—C5	121.6 (6)
C2—C3—H3	119.4	C3—C4—H4	119.2
C4—C3—C2	121.2 (5)	C5—C4—H4	119.2
C4—C3—H3	119.4	C13—C12—H12	119.5
C10—C15—H15	119.8	C11—C12—C13	121.0 (6)
C10—C15—C14	120.4 (6)	C11—C12—H12	119.5
C14—C15—H15	119.8	C5—C8—H8A	109.5
Se1—C1—H1A	109.0	C5—C8—H8B	109.5
Se1—C1—H1B	109.0	C5—C8—H8C	109.5
C2—C1—Se1	112.9 (4)	H8A—C8—H8B	109.5
C2—C1—H1A	109.0	H8A—C8—H8C	109.5
C2—C1—H1B	109.0	H8B—C8—H8C	109.5
H1A—C1—H1B	107.8	C13—C16—H16A	109.5
Se2—C9—H9A	109.0	C13—C16—H16B	109.5
Se2—C9—H9B	109.0	C13—C16—H16C	109.5
C10—C9—Se2	112.7 (4)	H16A—C16—H16B	109.5
C10—C9—H9A	109.0	H16A—C16—H16C	109.5
C10—C9—H9B	109.0	H16B—C16—H16C	109.5
			
Se1—Se2—C9—C10	−51.8 (5)	C1—Se1—Se2—C9	88.1 (3)
Se2—Se1—C1—C2	59.1 (4)	C1—C2—C7—C6	178.8 (5)
C10—C15—C14—C13	−1.0 (9)	C1—C2—C3—C4	−178.8 (5)
C10—C11—C12—C13	0.1 (9)	C9—C10—C15—C14	−177.8 (5)
C6—C5—C4—C3	−0.8 (9)	C9—C10—C11—C12	178.2 (5)
C2—C3—C4—C5	0.1 (9)	C5—C6—C7—C2	0.0 (9)
C7—C6—C5—C4	0.7 (9)	C14—C13—C12—C11	0.4 (9)
C7—C6—C5—C8	−178.5 (5)	C11—C10—C15—C14	1.4 (8)
C7—C2—C3—C4	0.6 (8)	C11—C10—C9—Se2	−78.6 (6)
C7—C2—C1—Se1	−97.9 (6)	C12—C13—C14—C15	0.1 (9)
C3—C2—C7—C6	−0.7 (8)	C8—C5—C4—C3	178.5 (6)
C3—C2—C1—Se1	81.6 (6)	C16—C13—C14—C15	−180.0 (6)
C15—C10—C9—Se2	100.6 (6)	C16—C13—C12—C11	−179.6 (6)
C15—C10—C11—C12	−0.9 (9)		

## References

[bb1] Agilent (2010). *CrysAlis PRO* Agilent Technologies, Yarnton, Oxfordshire, England.

[bb2] Garud, D. R., Koketsu, M. & Ishihara, H. (2007). *Molecules*, **12**, 504–535. 10.3390/12030504 PMC6149403 17851407

[bb3] Hua, G., Fuller, A. L., Slawin, A. M. Z. & Woollins, J. D. (2010). *Acta Cryst.* E**66**, o2579. 10.1107/S1600536810036676 PMC2983184 21587561

[bb4] Liu, W.-J., Wu, M.-H., Bao, C.-Y., Zou, W.-D. & Meng, X.-Y. (2006). *Acta Cryst.* E**62**, o3177–o3178.

[bb5] Saravanan, V., Porhiel, E. & Chandrasekaran, S. (2003). *Tetrahedron Lett.* **44**, 2257–2260.

[bb6] Sheldrick, G. M. (2008). *Acta Cryst.* A**64**, 112–122. 10.1107/S0108767307043930 18156677

[bb7] Westrip, S. P. (2010). *J. Appl. Cryst.* **43**, 920–925.

[bb8] Zhou, H., Ou, S.-Y., Yan, R.-A. & Wu, J.-Z. (2011). *Acta Cryst.* E**67**, o1938. 10.1107/S1600536811025736 PMC3212325 22090982

